# Anti‐aging drugs reduce hypothalamic inflammation in a sex‐specific manner

**DOI:** 10.1111/acel.12590

**Published:** 2017-05-20

**Authors:** Marianna Sadagurski, Gillian Cady, Richard A. Miller

**Affiliations:** ^1^ Division of Geriatric and Palliative Medicine Department of Internal Medicine University of Michigan Ann Arbor MI USA; ^2^ Department of Pathology and Geriatrics Center University of Michigan Ann Arbor MI USA; ^3^ Present address: Department of Biological Sciences Integrative Biosciences Center (IBio) Wayne State University Detroit MI USA

**Keywords:** Acarbose, aging, hypothalamus, inflammation, NDGA, longevity, sexual dimorphism, 17‐α Estradiol

## Abstract

Aging leads to hypothalamic inflammation, but does so more slowly in mice whose lifespan has been extended by mutations that affect GH/IGF‐1 signals. Early‐life exposure to GH by injection, or to nutrient restriction in the first 3 weeks of life, also modulate both lifespan and the pace of hypothalamic inflammation. Three drugs extend lifespan of UM‐HET3 mice in a sex‐specific way: acarbose (ACA), 17‐α‐estradiol (17αE2), and nordihydroguaiaretic acid (NDGA), with more dramatic longevity increases in males in each case. In this study, we examined the effect of these anti‐aging drugs on neuro‐inflammation in hypothalamus and hippocampus. We found that age‐associated hypothalamic inflammation is reduced in males but not in females at 12 months of age by ACA and 17αE2 and at 22 months of age in NDGA‐treated mice. The three drugs blocked indices of hypothalamic reactive gliosis associated with aging, such as Iba‐1‐positive microglia and GFAP‐positive astrocytes, as well as age‐associated overproduction of TNF‐α. This effect was not observed in drug‐treated female mice or in the hippocampus of the drug‐treated animals. On the other hand, caloric restriction (CR; an intervention that extends the lifespan in both sexes) significantly reduced hypothalamic microglia and TNF‐α in both sexes at 12 months of age. Together, these results suggest that the extent of drug‐induced changes in hypothalamic inflammatory processes is sexually dimorphic in a pattern that parallels the effects of these agents on mouse longevity and that mimics the changes seen, in both sexes, of long‐lived nutrient restricted or mutant mice.

**Abbreviations**
17αE2: 17‐α‐estradiol
ACA: acarbose
ARH: arcuate nucleus of the hypothalamus
CNS: central nervous system
CR: calorie restriction
NDGA: nordihydroguaiaretic acid

## Introduction

Increased inflammatory activity accompanies normal brain aging. Aging retardation can be achieved in mice by inhibiting activation of hypothalamic nuclear factor‐kB (NF‐kB) inflammatory pathways (Zhang *et al*., 2013). Similarly, limiting nutrient availability in the first 3 weeks of life (the crowded litter (CL) model) leads to reduced hypothalamic gliosis in parallel with reduced activation of tumor necrosis factor (TNF‐α) in the aged CL mice (Sadagurski *et al*., 2015a). Furthermore, the mutations that interfere with GH production or response, in the long‐lived Snell dwarf, Ames dwarf or growth hormone receptor deficient (GHRKO) mice lead to reduced age‐associated hypothalamic inflammation in old mice (Sadagurski *et al*., 2015b). Recent studies by the NIA Interventions Testing Program (ITP) demonstrated that lifespan can also be increased in male UM‐HET3 mice exposed to three drugs: acarbose (ACA), nordihydroguaiaretic acid (NDGA), and 17‐α‐estradiol (17αE2; Harrison *et al*., 2013). The genetically heterogeneous UM‐HET3 mice are the ideal model system to study lifespan, because the tested populations are reproducible and represent great genetic diversity (Miller *et al*., 1999, 2011). NDGA and 17aE2 do not extend lifespan of female UM‐HET3 mice, and the effect of ACA, although statistically significant in both sexes, is dramatically larger in males than in females (Harrison *et al*., 2013). Neither the site(s) of action responsible for these longevity effects, nor the basis for the sex specificity, are known.

It is well documented that many aspects of carbohydrate and energy metabolism, including pancreatic function and insulin action on the liver and other target organs, are influenced by the hypothalamus via neuronal signals (Myers, 2006) and that obesity, insulin resistance and metabolic syndrome are associated with hypothalamic inflammation (Morton *et al*., 2006; Thaler *et al*., 2012). Glial cells are particularly sensitive to homeostatic imbalances, and increased glial activity is a well‐established sign of an inflammatory response (Arvin *et al*., 1996). Activation of NF‐kB dependent cytokine production and hypothalamic glial activity increases with age, and augmentation of this effect can shorten mouse lifespan (Zhang *et al*., 2013), while blunting hypothalamic inflammatory signals can increase lifespan in mice (Zhang *et al*., 2013). Glial activity and production of mRNA for the inflammatory cytokine TNF‐α are diminished in long‐lived, GH‐deficient Ames and Snell dwarf mice, and in GHRKO mice (Sadagurski *et al*., 2015b), but not in mice with liver‐specific disruption of GHR, a mutant that does not show increased longevity (List *et al*., 2014).These observations are all consistent with the idea that lower hypothalamic inflammation may modulate aging processes, and lifespan, in mice.

Increased neuro‐inflammatory reaction is frequently observed not only in hypothalamus, but also in the hippocampus during normal brain aging (Gavilan *et al*., 2007). Age‐related hippocampal inflammatory processes are potentially related to hippocampal neurodegeneration (Mattson & Magnus, 2006). The aging hippocampus does not appear to suffer a generalized loss of cells, although atrophy of the structure may occur in humans, and these changes are thought to be an important contributor to age‐related cognitive impairments (Miller & O'Callaghan, 2005). Activated microglial cells are observed in the hippocampus of aged animals (Lynch *et al*., 2010), and high levels of several cytokines such as IL‐1β and TNF‐α, produced by the activated microglia, are significantly increased in hippocampus of aged mice and rats (Ye & Johnson, 1999; Norden & Godbout, 2013).

ACA is an inhibitor of intestinal α‐glucosidase (Balfour & McTavish, 1993). It inhibits digestion of polysaccharides and delays the uptake of sugars from the GI tract. It lowers postprandial glucose excursions and is used clinically for the treatment of type 2 diabetes (DiNicolantonio *et al*., 2015). In UM‐HET3 mice, ACA treatment beginning at 4 months of age increased median and maximal lifespan in males much more strongly than in females (Harrison *et al*., 2013). When started at 16 months, ACA significantly increased median longevity in males and 90th percentile lifespan in both sexes (Strong *et al*., 2016). ACA reduced fasting insulin significantly in young males but not in females, while the reduction in body weight and age‐associated decline in voluntary activity was more dramatic in ACA‐fed females than in males (Harrison *et al*., 2013).

17αE2 is an optical isomer of 17‐β‐estradiol that has reduced affinity for estrogen receptors (Zhurova *et al*., 2009). This form of estrogen is reported to be neuroprotective *in vitro* in cultured cells and *in vivo* in an ischemia–reperfusion animal model (Perez *et al*., 2005). It also has been reported to protect against neurodegeneration in cell and animal models of Parkinson's disease (Dykens *et al*., 2005) and cerebrovascular disease (Liu *et al*., 2005). An initial report using 17aE2 at 4.8 ppm showed increased median and maximal lifespan in males but no effects on lifespan in female mice (Harrison *et al*., 2013). A second study using 17αE2 at 14.4 ppm robustly extended both median and maximal lifespan in males, but showed no effect in females, and 17aE2‐treated males lived significantly longer than either control or treated females (Strong *et al*., 2016).

NDGA is both a lipoxygenase inhibitor and potent antioxidant (Lu *et al*., 2010). Two ITP studies have shown that NDGA increases median lifespan, but only in male mice (Strong *et al*., 2008; Harrison *et al*., 2013). The lack of effect of NDGA on female lifespan cannot be explained by effects on body weight (Miller *et al*., 2007; Harrison *et al*., 2013) or by differences in blood NDGA concentrations. NDGA inhibits inflammatory responses that are thought to play a role in many age‐related diseases (Ferrandiz & Alcaraz, 1991).

Changes in gene expression and glial localization in the course of normal brain aging are sexually dimorphic (Berchtold *et al*., 2008). For example, males have more microglia early in postnatal development, whereas females have more microglia with an activated morphology later in life, as young adults (Schwarz *et al*., 2012). Furthermore, the numbers and diversity of astrocytes are also sexually differentiated across brain regions (McCarthy & Arnold, 2011). Gene expression of a large number of cytokines in the aging brain is highly dependent upon sex and region (Bilbo *et al*., 2012). These collective data suggest that regional‐, sex‐, and age‐dependent differences in glia cells and their inflammatory capacity could play a role in the sex‐specific lifespan benefit of ACA, 17αE2, and NDGA.

In this study, we evaluated the effect of ACA, 17αE2, and NDGA on age‐associated inflammatory responses in two brain areas that are sensitive to aging and age‐related neurological changes (hypothalamus and hippocampus), to determine whether these are modified by the anti‐aging drugs and whether the effects of the drugs on inflammation differ in males and females.

## Results

### Effect of ACA and 17αE2 on CNS inflammation in aged mice

We evaluated two indices of inflammatory responses, that is, glial fibrillary acidic protein (GFAP, a marker of astrocyte activation), and production of TNF‐α by microglia, in the hypothalamus and hippocampus of 12‐month‐old UM‐HET3 male and UM‐HET3 female mice that had been exposed to ACA or 17αE2 from 4 months of age.

**Figure 1:**
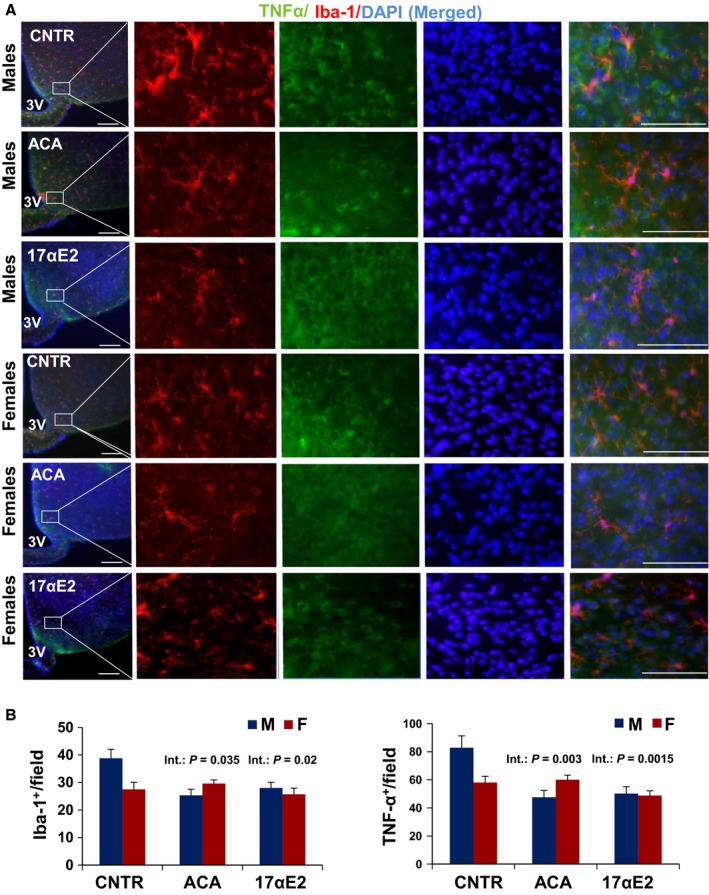
Hypothalamic inflammation in ACA‐ and 17αE2‐treated mice as measured by Iba‐1^+^ microgial and TNF‐α cells. Brain sections of 12‐month‐old male and female mice were analyzed for hypothalamic microglia and TNF‐α. (A) Representative images showing immunostaining in the MBH of control ACA‐ and 17αE2‐treated mice. Scale bars: 100 μm (far left); 20 μm (right side panels), 3V, third ventricle. (B) Numbers of cells immunoreactive for Iba‐1 or TNF‐α in the hypothalamic mediobasal region (across the confocal microscopic field of serial sections) from indicated male and female mice; error bars show SEM for *N* = 6 mice of each type. The p‐value shown as ‘Int’ represents the interaction term in a two‐factor ANOVA, testing whether the drug effect differs between male and female mice.

**Figure 2:**
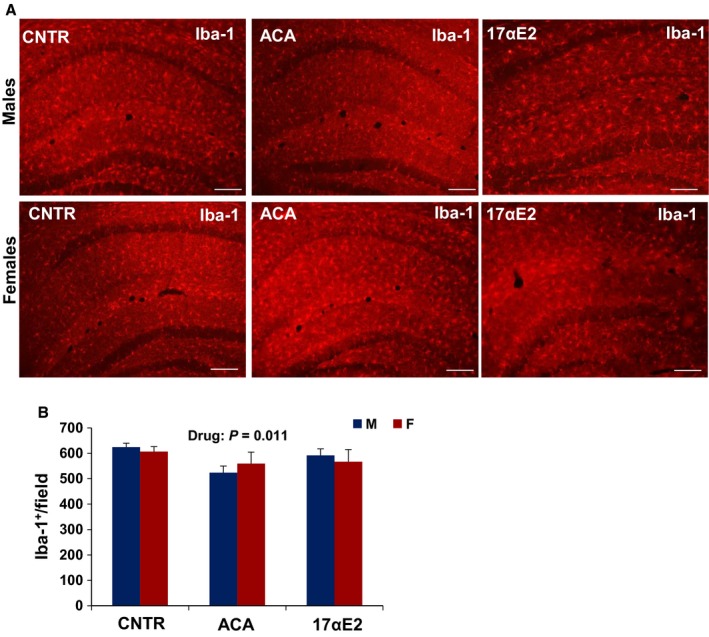
Microglia in the hippocampus of ACA‐ and 17αE2‐treated mice. (A) Representative images of microglia identified by immunofluorescent detection of Iba‐1 protein in coronal sections of CA1, CA3, and dentate gyrus of the hippocampus obtained from 12‐month‐old male and female control, ACA‐, and 17αE2‐treated mice. Scale bars: 100 μm (B) Quantification of Iba‐1 in coronal sections of left CA1, CA3, and dentate gyrus considered together. Bars show mean and SEM for *N* = 6 mice of each type. Acarbose (ACA) led to a significant decline (*P* = 0.011) in Iba‐1^+^ cells, and there was no significant interaction effect for both treatments.

We used Iba‐1 as a marker of microglial number and analyzed results by analysis of variance to test the main effects of sex and drug exposure and the [sex × drug] interaction term. ACA had a significant effect (*P* = 0.01) reducing activated microglia in both hypothalamus and hippocampus, with no significant main effect of sex (*P* = 0.07 for MBH and *P* = 0.7 for hippocampus; Figs 1 and 2A,B). The interaction term was significant at *P* = 0.035 for hypothalamus, showing that the drug had greater effects on microglial numbers in males than in females (Fig. 1B). There was no evidence for sex specificity (*P* = 0.33) in hippocampus (Fig. 2A,B).

Previous studies by us and others have demonstrated that about 80% of the hypothalamic Iba1^+^ cells produced tumor necrosis factor‐α (TNF‐α), indicating that they are inflammatory (Zhang *et al*., 2013; Sadagurski *et al*., 2015a). Analysis of variance revealed a significant effect of drug (*P* = 0.008) and a significant interaction of sex and drug on TNF‐α expression in the MBH (*P* = 0.003), without a main effect of sex (*P* = 0.29; Fig. 1). Thus, ACA diminishes this aspect of inflammation more dramatically in male than in female mice, with effects demonstrable as early as 12 months of age. We were not able to reliably detect TNF‐α by immunostaining in the hippocampus.

**Figure 3:**
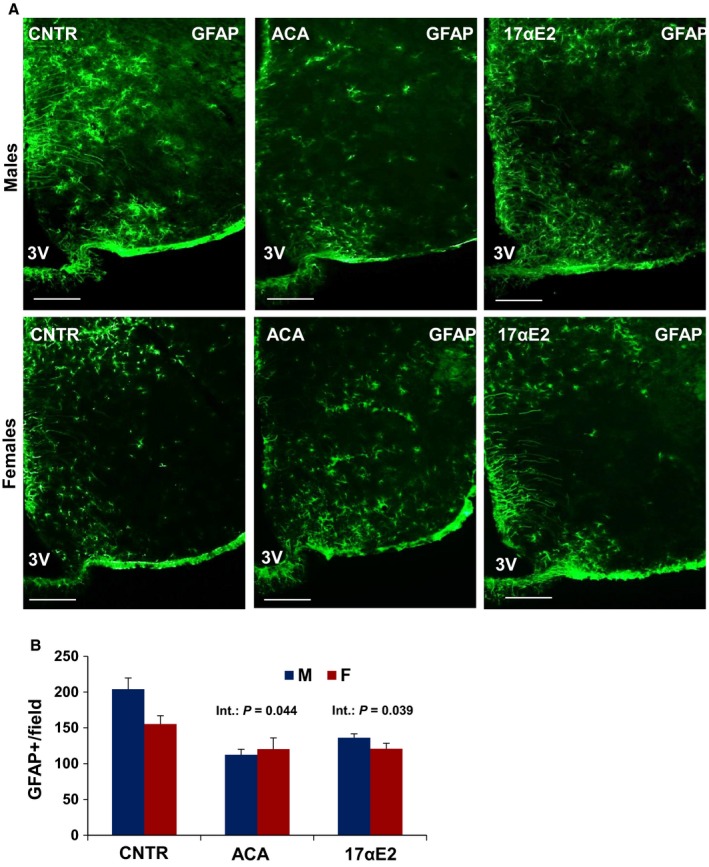
Hypothalamic astrogliosis in ACA‐ and 17αE2‐treated mice. (A) Representative images of astrocytes identified by immunofluorescent detection of GFAP protein in coronal sections of hypothalamus obtained from 12‐month‐old male and female control, ACA‐, and 17αE2‐treated mice. Scale bar: 100 μm. 3V, third ventricle. (B) Quantification of GFAP staining represents number of GFAP‐positive cells per field (error bars indicate SEM;* N* = 6 mice per group) in the MBH subregion. The significant interaction *P*‐value (*P* = 0.044) for ACA and (*P* = 0.039) for 17αE2 indicates that both drugs have different effects on GFAP ^+^ cells in males vs. females.

**Figure 4:**
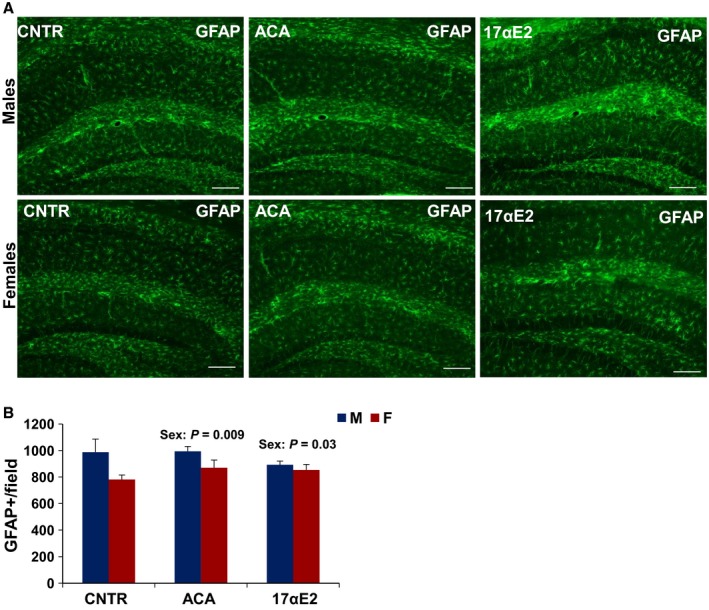
Astrocytes in the hippocampus of ACA‐ and 17αE2‐treated mice. (A) Representative images of astrocytes identified by immunofluorescent detection of GFAP protein in coronal sections of CA1, CA3, and dentate gyrus of the hippocampus obtained from 12‐month‐old male and female control, ACA‐, and 17αE2‐treated mice. Scale bars: 100 μm (B) Quantification of GFAP staining represents number of GFAP‐positive cells in coronal sections of left CA1, CA3, and dentate gyrus considered together. Bars show mean and SEM for *N* = 6 mice of each type. There was no significant interaction effect for GFAP with either drug. GFAP ^+^ cells were significantly higher in male mice regardless of drug treatment.

17αE2 treatment had effects similar to those of ACA on hypothalamic microglia (Fig. 1). In male mice evaluated at 12 months of age, 17αE2 diminished hypothalamic microglia (*P* = 0.02), with significant interaction terms (*P* = 0.02). In addition, the ANOVA revealed a significant effect of 17αE2 (*P* = 0.001) and a significant interaction of sex and drug (*P* = 0.0015) on TNF‐α expression in the MBH of 12‐month‐old 17αE2‐treated animals (Fig. 1). For none of these assays was there a significant main effect of sex per se (Fig. 1). A parallel analysis of the hippocampus showed no effect of 17aE2 (*P* = 0.83) and no interaction between 17aE2 and sex (*P* = 0.13; Fig. 2A,B). Astrogliosis with advancing age is correlated with increased expression of (GFAP; Nichols *et al*., 1995). The number of GFAP‐positive astrocytes in hypothalamus was higher in male control than in female control (*P* = 0.03) and was diminished by both ACA (*P* < 0.0001) and 17αE2 (*P* = 0.001), with significantly more effect in male than in female mice (interaction *P* = 0.044 for ACA and *P* = 0.039 for 17αE2; Fig. 3A,B).The number of GFAP‐positive astrocytes in the hippocampus depended on sex at 12 months of age (*P* = 0.009, with males demonstrating higher GFAP‐positive cell at this age), but there was no significant effect of ACA (*P* = 0.42) or 17αE2 (*P* = 0.83) and no significant interaction term in the hippocampus (for ACA, *P* = 0.48 and for 17αE2, *P* = 0.13; Fig. 4A,B).

We detected no significant effects of sex or treatment in the total number of cells detected by DAPI staining (data not shown).

In support of these findings, two‐way ANOVA revealed a significant effect of caloric restriction (an intervention that extends the lifespan in both sexes) on both hypothalamic microglia (*P* = 0.0001) and TNF‐α (*P* = 0.0001) without significant interaction of sex and drug for both endpoints at 12 months of age (Fig. S1A,B, Supporting information). The effect of CR on GFAP‐positive astrocytes in the hypothalamus did not reach statistical significance (*P* = 0.09; Fig. S1C,D, Supporting information). Consistent with the hippocampal results for 12‐month‐old ACA and 17αE2‐treated mice, the number of GFAP‐positive astrocytes in the hippocampus depended on sex (*P* = 0.002), but there were no significant effects of CR on microglia (*P* = 0.35) or number of astrocytes (*P* = 0.07; Fig. S1E–H, Supporting information).

### Effect of NDGA on CNS inflammation in old mice

**Figure 5:**
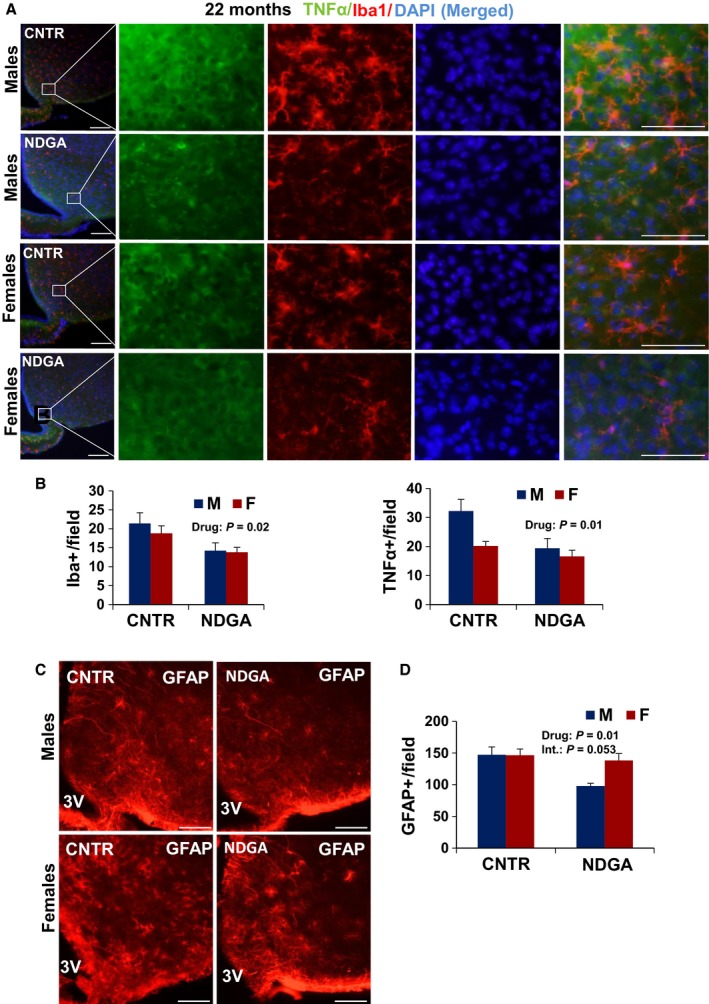
NDGA diminishes hypothalamic inflammation in hypothalamus. Brain sections of 22‐month‐old male and female mice were analyzed for hypothalamic microglia, TNF‐α, and astrocytes. (A) Representative images showing Iba‐1 and TNF‐α immunostaining merged with DAPI (blue) in the hypothalamic mediobasal region (MBH) of control and NDGA‐treated mice. Scale bars: 100 μm (far left); 20 μm (right side panels), 3V, third ventricle. (B) Numbers of cells immunoreactive for Iba‐1 or TNF‐α in the MBH (across the confocal microscopic field of serial sections) from indicated male and female mice. (C) Representative images of astrocytes identified by immunofluorescent detection of GFAP protein in coronal sections of hypothalamus. Scale bar: 100 μm. 3V, third ventricle. (D) Quantification of GFAP staining represents number of GFAP‐positive cells per field in the MBH subregion. Bars show mean and SEM for 4 or 5 mice/group. Two‐factor ANOVA showed a significant effect of drug on microglia numbers and TNF‐α, but no significant interaction (*P* = 0.14 for TNF‐α cells) between sex and drug treatment. NDGA led to a significant reduction (*P* = 0.01) in GFAP ^+^ cells, and the [sex × drug] interaction term just failed to reach significance at *P* = 0.053.

**Figure 6:**
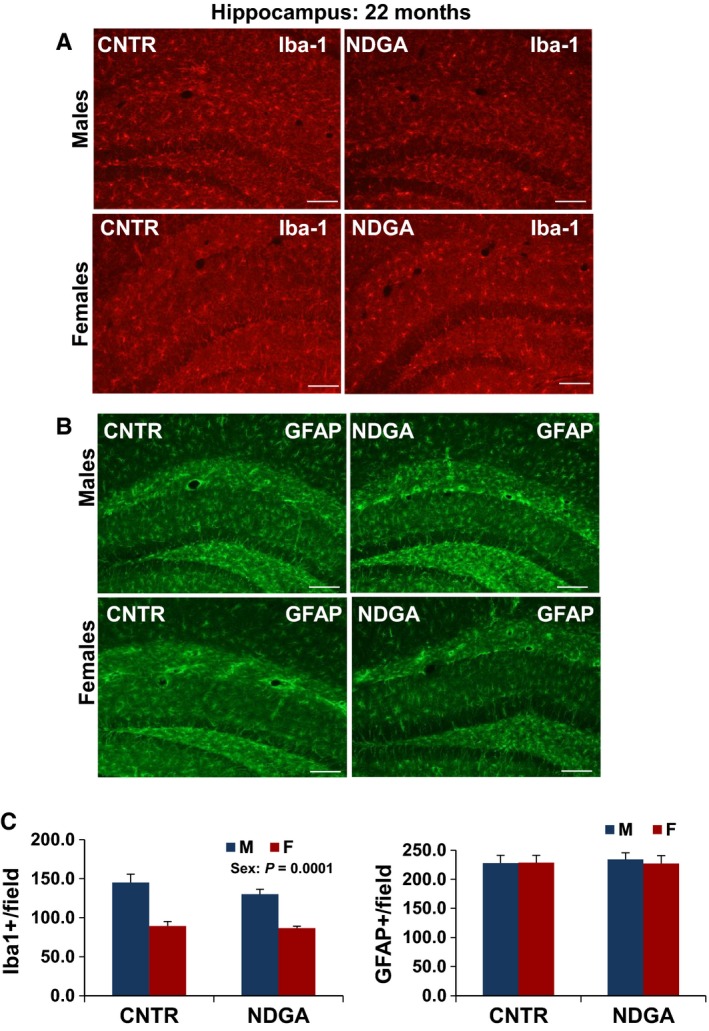
Microglia and astrocytes in the hippocampus of 22‐month‐old NDGA‐treated mice. (A) Representative images of microglia identified by immunofluorescent detection of Iba‐1 protein and (B) astrocytes identified by immunofluorescent detection of GFAP protein in coronal sections of CA1, CA3, and dentate gyrus of the hippocampus obtained from 22‐month‐old male and female NDGA‐treated mice, scale bar: 100 μm. (C) Quantification of Iba‐1 and GFAP staining represents number of Iba‐1 or GFAP‐positive cells in coronal sections of left CA1, CA3, and dentate gyrus. Bars show mean and SEM for *N* = 6. Iba‐1^+^ cells were higher in males (*P* = 0.0001).

The anti‐inflammatory agent NDGA leads to a male‐specific extension of median lifespan. The effects of NDGA are dose dependent but without an effect on maximal lifespan in either sex (Harrison *et al*., 2013; Strong *et al*., 2016). Our previous work has shown continuing age‐related increase in levels of hypothalamic inflammatory markers (TNF‐α, Iba‐1, and GFAP) between 12 and 22 months in UM‐HET3 mice (Sadagurski *et al*., 2015a). At 22 months of age, we found reduced numbers of GFAP‐immunostained astrocytes and Iba‐1 microglia in the MBH of NDGA‐treated mice (*P* < 0.01) as compared to control mice (Fig. 5). For astrocytes, the (sex × drug) interaction term, *P* = 0.052, did not quite reach conventional levels for statistical significance, nor was there evidence for significant sex specificity in the microglial count (*P* = 0.4; Fig. 5). Similarly, we observed a significant effect of NDGA (*P* = 0.013) and sex (*P* = 0.023) on TNF‐α expression in hypothalamic microglia, without a significant interaction between NDGA treatment and sex (Fig. 5A,B). Lastly, we saw no effect of NDGA treatment on astrocytes and microglia activation in the hippocampus of 22‐month‐old mice (Fig. 6).

## Discussion

These data are the first to document the effect of lifespan‐extending drugs on neuro‐inflammatory components, within brain regions that are sensitive to aging. Modulation of lifespan by NDGA, ACA, and 17aE2 is sex specific, with males being more responsive to each of these agents (Strong *et al*., 2008, 2016; Harrison *et al*., 2013). We demonstrate here that hypothalamic inflammatory responses in 22‐month‐old NDGA and 12‐month‐old ACA and 17αE2‐treated mice are reduced by these drugs in males but not in females. We saw no consistent effects on inflammation in the hippocampus of the drug‐treated mice, supporting the idea that inhibition of the hypothalamic inflammatory responses may play a particularly important role in mouse lifespan extension (Zhang *et al*., 2013). Similarly, early‐life caloric restriction (CL between birth and 3 weeks of age; Sadagurski *et al*., 2015a) and mutations that interfere with GH production or response (Sadagurski *et al*., 2015b) also lead to reduced hypothalamic inflammation in old mice. Our data now provide evidence that drugs that extend lifespan might be effective in inhibiting hypothalamic inflammatory processes in a sex‐dependent manner.

It seems likely that the sex‐specific effects of these drugs on both inflammation and lifespan may reflect differences in hormonal patterns between the sexes, although our current data do not distinguish between effects of sex hormones in early life (i.e., prior to drug exposure) and possible interactions with endocrine status during the period of drug treatment. Neuron–glial plasticity is heavily influenced by sex steroids and contributes to differential immune responses by sex (Zagni *et al*., 2016). Sex steroids regulate the transcription of genes, such as cytokines relevant to immune response and immune signaling (Barna *et al*., 1996). In our study, we did not detect any effects of sex or drug treatment on total cell numbers (DAPI staining), but we cannot exclude the possibility that there may be have effects on neuronal cell numbers. Future studies of the effects of ACA, 17αE2, and NDGA in ovariectomized females and castrated males, or in mice lacking the classical estrogen receptor, or in testosterone‐treated females, would help to clarify the basis for the effects of these drugs on male mice. Information from such studies might suggest ideas about how the health benefits of these three drugs might be extended to female mice and about approaches to development of drugs that postpone aging both in men and women.

In several of the models we have evaluated, drug treatment diminishes inflammation in male mice to the level seen in untreated female mice, but has little effect on the female animals. This observation suggests the possibility of a threshold effect, the idea that inflammation in females may already be at such a low level that drug treatment cannot lead to further diminution. There is substantial evidence for sex‐specific differences in both microglia and astrocytes in the mouse brain (McCarthy & Arnold, 2011; Pfau *et al*., 2016). Males have more microglia in the CA1, CA3, and dentate gyrus (DG) of the hippocampus and the amygdala early in postnatal development, whereas females have more microglia with an activated morphology later in development, that is, as juveniles and as young 3‐month‐old adults (Schwarz *et al*., 2012). Our data show that male mice have higher levels of hypothalamic glia than females, consistent with limited data in monkeys (Roberts *et al*. 2012), changes that may reflect early developmental effects rather than effects of aging. Sex differences in glial number and morphology depend on brain region and may well be modulated differently by drug and endocrine status at later ages as well. We found no significant effect of the anti‐aging drugs on microglia numbers in the hippocampus at 12 or 22 months of age, although females had less microglia than males at the older age. Consistent with this observation, we found no significant effect of the drugs on astrocyte cell counts in hippocampus. A more detailed analysis of separate subregions of the hippocampus would be laborious and would require sections cut at orientations optimized for this purpose, but might provide additional useful detail about regional heterogeneity. Drugs that extend lifespan equally in males and females, or which render females more responsive to the lifespan effects mediated by ACA and 17αE2, will help provide additional insight into this sexual dimorphism.

In contrast to our data, a recent study by Hascup *et al*. (2016), examining mRNA expression levels, found significant elevation in certain NF‐kB signaling molecules and glutamatergic markers in the hypothalamus and different hippocampus areas in old GHR−/− mice as compared to controls. The methods used by Hascup provided relatively little information about the specific cell type(s) expressing NF‐kB signaling molecules. In contrast, the conclusions of Zhang (Zhang *et al*., 2013) and from our current and previous studies (Sadagurski *et al*., 2015b) are based on immunostaining evaluation of inflammatory markers. Such assessment provides an anatomical location of cells within the area of interest suggesting the overall function to which those cells contribute. As differential physiological properties have been reported across hypothalamic areas as well as across septal and temporal hippocampus, a combined approach of comprehensive molecular mapping of inflammatory genes based on *in situ* hybridization (ISH) data in long‐lived mice can provide additional insight into discrete cellular populations.

Glial cells are particularly sensitive to homeostatic imbalances. Nutritional excess is a key activator of metabolic inflammation in the hypothalamus, suggesting that interventions that can counteract hypothalamic neuro‐inflammation may protect against metabolic disorders (Thaler & Schwartz, 2010). An inflammatory state in the hypothalamus disrupts its ability to sense metabolic abnormalities, affecting energy balance and glucose metabolism, leading to obesity and insulin resistance (Thaler *et al*., 2012). It was recently demonstrated that there is a sexually dimorphic response to chronic high‐fat diet (HFD) exposure (Morselli *et al*., 2014). Consistent with some of our findings in aged mice, the work on HFD demonstrated that males, but not females, have hypothalamic inflammation despite comparable weight gain following HFD consumption (Morselli *et al*., 2014), further emphasizing the physiological relevance of hypothalamic inflammation.

The sphingolipid system has emerged as a key player in modulation of microglia phenotype in pathIogenic forms of neuroinflammation (Assi *et al*., 2013). Sphingolipids accumulate in the liver and brain during aging, and long‐term food restriction prevents aging‐associated sphingolipid turnover dysregulation (Babenko & Shakhova, 2014). Elevated levels of sphingolipids have previously been associated with reduced insulin signaling and increased inflammation within the CNS (Summers, 2006). Sphingolipids were significantly increased in the hypothalamus of male animals fed with a HFD when compared to the females (Morselli *et al*., 2014). The recent study by the Gladyshev group showed that brains of old mice had higher concentrations of glycerophospholipids and sphinogmyelins (Ma *et al*., 2015), key constituents of cell membranes which play a role in neuroinflammation (Yadav & Tiwari, 2014). Future metabolomics analysis from the hypothalamus per se, from normal and drug‐treated mice, would help to elucidate the underlying biochemical mechanisms of sex‐specific drug effects on hypothalamic inflammation in aging.

In previously published work (Harrison *et al*., 2013), fasting insulin levels in males were higher than in female controls, and ACA reduced insulin levels in males to the level of control females. The male‐specific decline in fasting insulin level in ACA‐treated mice hints that the altered pattern of glucose spikes produced by ACA may lead to improved insulin sensitivity in males, with less effect in females, and that this protection against insulin resistance might in turn contribute to the sexual dimorphism in longevity effect (Harrison *et al*., 2013) and in the age‐associated hypothalamic inflammation demonstrated in this study. It has been reported that reduction in postprandial glucose peak levels by ACA in patients with type 2 diabetes reduces postprandial activation of the NF‐κB pathway in blood cells (Rudofsky *et al*., 2004). NDGA lowers blood glucose, ameliorates hypertriglyceridemia in male rats (female rats were not tested; Zhang *et al*., 2015), and interferes with TNF‐induced NF‐κB‐mediated transactivation in 293 cells (van Puijenbroek *et al*., 1999). The activity of ACA, NDGA, and 17αE2 as anti‐inflammatory agents opens new avenues for mechanistic testing and will prove to be valuable for studying differences between male and female mice for control of aging and late‐life neurological diseases. It would be of interest to learn whether ACA, NGDA, or 17αE2 modulates additional pathways thought to be relevant to aging and lifespan, such as those linked to ATF4, mTOR, autophagy, adipokine production, proteasome function, and others, and do so in a sex‐specific way.

## Methods

### Animals

Procedures involved in this study were approved by the University of Michigan Committee on the Use and Care of Animals (UCUCA). UM‐HET3 mice were produced as previously described in detail (Miller *et al*., 2007). For breeding cages, we used Purina 5008 mouse chow. For weanlings prior to 4 months of age, we used Purina 5LG6. Animals were maintained under temperature‐ and light‐controlled conditions (20–23 °C, 12‐h light–dark cycle).

### Experimental diets

17αE2 was purchased from Steraloids Inc. (Newport, RI, USA) and mixed at a dose of 14.4 mg kg^−1^ diet (14.4 ppm). Mice were fed the 17αE2 diet continuously from 4 months of age. NDGA was purchased from Cayman Chemicals (Ann Arbor, MI, USA) and mixed at a concentration of 2500 ppm and fed to mice beginning at 4 months of age. ACA was purchased from Spectrum Chemical Mfg. Corp., Gardena, CA, USA. It was mixed at a concentration of 1000 mg of ACA per kilogram of diet (1000 ppm); mice were fed continuously from 4 months of age. For CR feeding: starting at 4 months of age, CR mice were given 80% of the amount of food consumed by age‐matched ad lib control mice for 2 weeks. After that and continuing until euthanasia at 12 months of age, the CR mice were given 60% of diet consumed by ad lib control mice, as previously described (Miller *et al*., 2014).

### Perfusion and immunolabeling

Mice were anesthetized (IP) with Avertin and transcardially perfused with phosphate‐buffered saline (PBS; pH 7.5) followed by 4% paraformaldehyde (PFA). Brains were postfixed, dehydrated, and then sectioned coronally (30 μm) using a sliding microtome, followed by immunohistochemical or immunofluorescent analysis as previously described (Sadagurski *et al*., 2015b). Brains from 22‐month‐old control and NDGA‐treated mice were only postfixed with 4% PFA. For immunohistochemistry, free‐floating brain sections were pretreated by sequential incubations in 0.3% H_2_O_2_/1% NaOH, 0.3% glycine, 0.03% SDS, followed by blocking in normal donkey serum (NDS). Sections were incubated with rabbit anti‐GFAP (1:1000; Millipore, Temecula, CA, USA), mouse anti‐TNF‐α (1:500; Abcam, Cambridge, MA, USA) or rabbit anti‐Iba‐1 (1:1000; Wako, Richmond, VA, USA) as primary antibodies followed by AlexaFluor‐conjugated secondary antibodies (Invitrogen, Carlsbad, CA, USA) as previously published (Sadagurski *et al*., 2012; Zhang *et al*., 2013).

Sections were mounted onto Superfrost Plus slides (Fisher Scientific, Hudson, NH, USA) and coverslips added with ProLong Antifade mounting medium (Invitrogen). Microscopic images were obtained using an Olympus FluoView 500 Laser Scanning Confocal Microscope (Olympus, Center Valley, PA, USA) equipped with a 10×, 20×, and 40× objectives.

### Quantification

For quantification of immunoreactive cells, images of matched brain areas were taken from at least three sections containing the MBH of the hypothalamus for each brain sections between bregma −0.82 mm to −2.4 mm (according to the Franklin mouse brain atlas). Serial brain sections across the hypothalamus were made at 30 μm thickness, and every five sections were represented by one section with staining and cell counting. For quantification of the hippocampus sections, left side of CA1, CA3, and dentate gyrus was considered together. All sections were arranged from rostral to caudal to examine the distribution of labeled glial cells. Iba‐1 and GFAP‐positive cells were counted using ImageJ software with DAPI (nuclear stain). The average of the total number of cells/field of view was used for statistical analysis as described previously (Sadagurski *et al*., 2015b).

### Statistical analysis

Data sets were analyzed using two‐way analysis of variance (ANOVA) followed by Tukey's post hoc test. All data were presented as mean ± SEM. *P *< 0.05 was considered significant. IBM SPSS v.21 was used for statistical analysis.

## Funding

This project was supported by NIA grants AG022303 and the Glenn Foundation for Medical Research. MS was supported by a pilot grant from the UM Pepper Center AG‐024824 and a Feasibility Grant from the Michigan Diabetes Research Center (P30DK020572).

## Conflict of interest

No conflict of interest, financial or otherwise, is declared by the authors.

## Supporting information


**Fig. S1** Microglia and astrocytes in the hypothalamus and hippocampus of CR treated mice.
