# Supplementary figures and images for: Anti‐aging drugs reduce hypothalamic inflammation in a sex‐specific manner
Source: Aging Cell. 2017 May 20;16(4):652–60. doi: 10.1111/acel.12590 (PMC5506421; doi:10.1111/acel.12590)

# Supplementary Figure 1

**A**

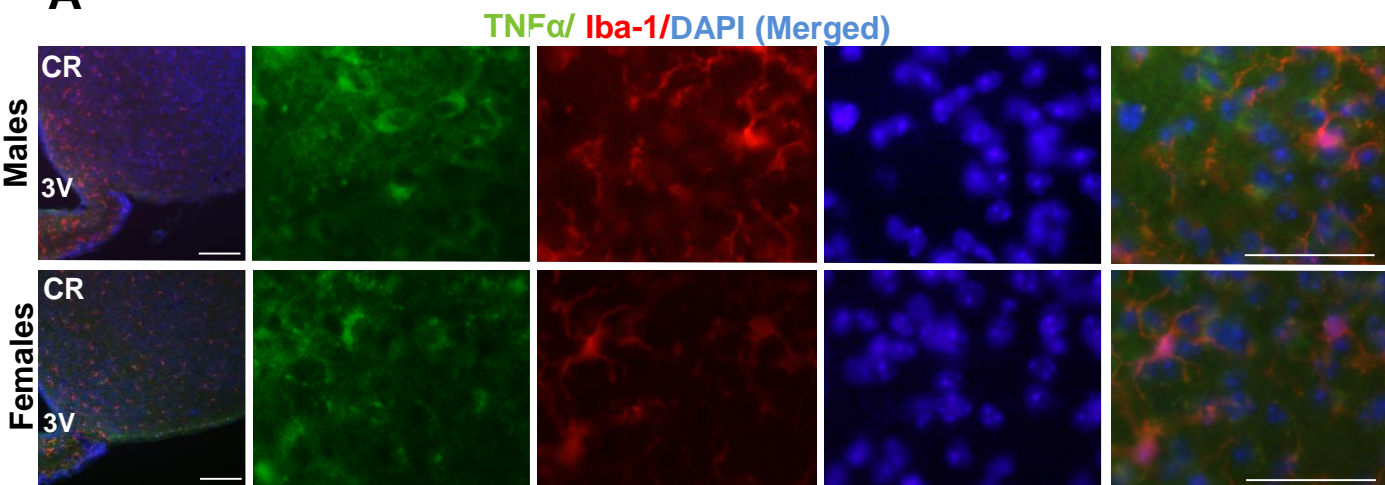

**B**

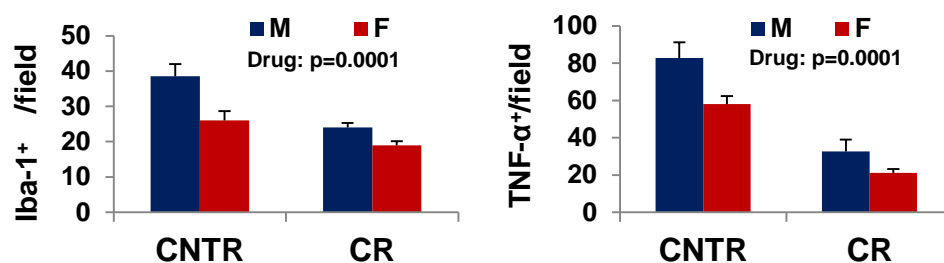

**C**

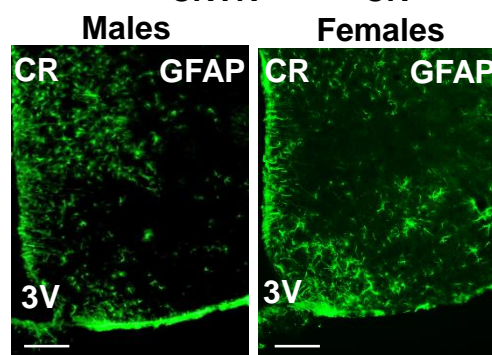

**D**

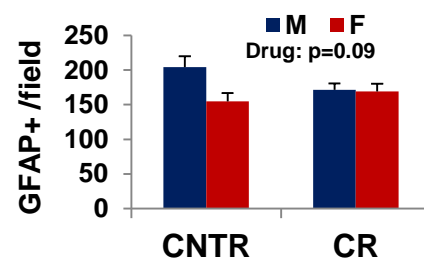

**E**

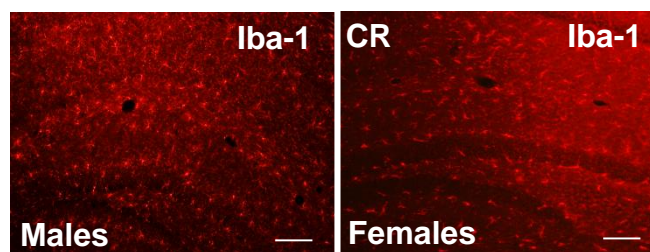

**F**

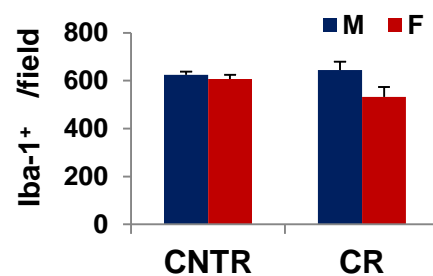

**G**

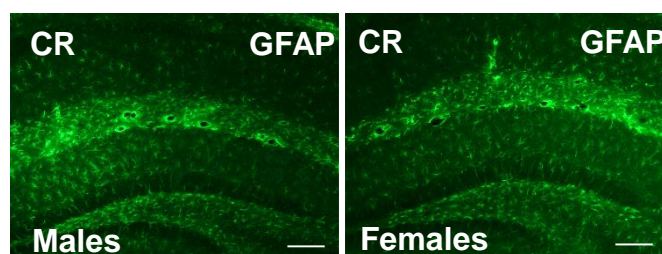

**H**

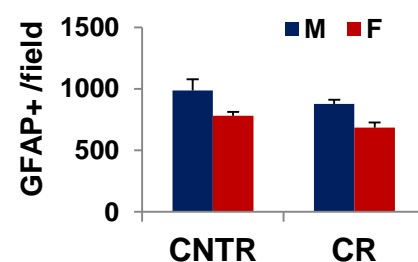

Supplement: Supplementary file 1 — Fig. S1 Microglia and astrocytes in the hypothalamus and hippocampus of CR treated mice. [file ACEL-16-652-s001.pdf]
