# Supplementary material for: Anti‐aging drugs reduce hypothalamic inflammation in a sex‐specific manner
Source: Aging Cell. 2017 May 20;16(4):652–60. doi: 10.1111/acel.12590 (PMC5506421; doi:10.1111/acel.12590)
Supplement: Supplementary file 2 [file ACEL-16-652-s002.docx]

**SUPPORTING INFORMATION**

**Anti-Aging Drugs Reduce Hypothalamic Inflammation in a Sex-Specific Manner**

^1^Marianna Sadagurski, ^2^Gillian Cady and ^2^Richard A. Miller

^1^Department of Internal Medicine, Division of Geriatric and Palliative Medicine

^2^Department of Pathology and Geriatrics Center, University of Michigan, Ann Arbor, MI

Running title: Anti-aging drugs and hypothalamic inflammation

Key words: hypothalamus; inflammation; Acarbose; NDGA; 17-α Estradiol; longevity; aging; sexual dimorphism

Corresponding author:

Marianna Sadagurski,

Department of Internal Medicine,

Division of Geriatric and Palliative Medicine

University of Michigan

Room 3003 BSRB,

109 Zina Pitcher Place, Ann Arbor, MI  48109-2200

Phone: (734) 936 2122

Fax: (734) 647 9749

Email: msadagur@med.umich.edu

**Legends:**

**Supplementary Figure 1:** Microglia and astrocytes in the hypothalamus and hippocampus of CR treated mice. (A) Representative images showing immunostaining in the MBH of CR treated mice. Representative control sections are shown in Main Figure 1. Scale bars: 100 μm (far left); 20 μm (right side panels), 3V, third ventricle. (B) Numbers of cells immunoreactive for Iba-1, or TNF-α in the hypothalamic mediobasal region (across the confocal microscopic field of serial sections) from indicated male and female mice; error bars show SEM for N = 6 mice of each type. The p-value shows a significant effect of caloric restriction on Iba-1 (p=0.0001) and TNFα (p=0.0001) without significant interaction of sex and drug shown treatment effect by two-factor ANOVA. (C) Microglia in the hippocampus of CR treated mice. Representative control sections are shown in Main Figure 2. (D) Iba-1 staining represents number of Iba-1 positive cells in coronal sections of left CA1, CA3 and dentate gyrus (n = 6 mice / group). (E) Representative images of astrocytes identified by immunofluorescent detection of GFAP protein in coronal sections of hypothalamus obtained from 12-month-old male and female CR treated mice. Representative control sections are shown in Main Figure 3. Scale bar: 100 µm. 3V, third ventricle. (F) Quantification of GFAP staining represents number of GFAP positive cells per field (error bars indicate SEM; N = 6 mice per group) in the MBH subregion. (G) Astrocytes in the hippocampus of CR treated mice. Representative control sections are shown in Main Figure 4. (H) Quantification of GFAP staining represents number of GFAP positive cells in coronal sections of left CA1, CA3 and dentate gyrus considered together (n = 6 mice / group). CR had no effect on astrocytes and number of activated microglia in the hippocampus of 12-month-old mice.
